# Community-acquired bacteraemia in COVID-19 in comparison to influenza A and influenza B: a retrospective cohort study

**DOI:** 10.1186/s12879-021-05902-5

**Published:** 2021-02-22

**Authors:** Julinha M. Thelen, A. G. ( Noud) Buenen, Marjan van Apeldoorn, Heiman F. Wertheim, Mirjam H. A. Hermans, Peter C. Wever

**Affiliations:** 1 Department of Medical Microbiology and Infection Control, Jeroen Bosch Hospital, ‘s-Hertogenbosch, the Netherlands; 2 Radboud University Nijmegen, Nijmegen, the Netherlands; 3 Department of Emergency Medicine, Bernhoven Hospital, Uden, the Netherlands; 4 Department of Internal Medicine, Jeroen Bosch Hospital, ‘s-Hertogenbosch, the Netherlands; 5 Department of Medical Microbiology, Radboud university medical center, Nijmegen, the Netherlands

## Abstract

**Background:**

During the coronavirus disease 2019 (COVID-19) pandemic in the Netherlands it was noticed that very few blood cultures from COVID-19 patients turned positive with clinically relevant bacteria. This was particularly evident in comparison to the number of positive blood cultures during previous seasonal epidemics of influenza. This observation raised questions about the occurrence and causative microorganisms of bacteraemia in COVID-19 patients, especially in the perspective of the widely reported overuse of antibiotics and the rising rate of antibiotic resistance.

**Methods:**

We conducted a retrospective cohort study on blood culture results in influenza A, influenza B and COVID-19 patients presenting to two hospitals in the Netherlands. Our main outcome consisted of the percentage of positive blood cultures. The percentage of clinically relevant blood cultures, isolated bacteria and 30-day all-cause mortality served as our secondary outcomes.

**Results:**

A total of 1331 viral episodes were analysed in 1324 patients. There was no statistically significant difference (*p* = 0.47) in overall occurrence of blood culture positivity in COVID-19 patients (9.0, 95% CI 6.8–11.1) in comparison to influenza A (11.4, 95% CI 7.9–14.8) and influenza B patients (10.4, 95% CI 7.1–13.7,). After correcting for the high rate of contamination, the occurrence of clinically relevant bacteraemia in COVID-19 patients amounted to 1.0% (95% CI 0.3–1.8), which was statistically significantly lower (*p* = 0.04) compared to influenza A patients (4.0, 95% CI 1.9–6.1) and influenza B patients (3.0, 95% CI 1.2–4.9). The most frequently identified bacterial isolates in COVID-19 patients were *Escherichia coli* (*n* = 2) and *Streptococcus pneumoniae* (n = 2). The overall 30-day all-cause mortality for COVID-19 patients was 28.3% (95% CI 24.9–31.7), which was statistically significantly higher (p = <.001) when compared to patients with influenza A (7.1, 95% CI 4.3–9.9) and patients with influenza B (6.4, 95% CI 3.8–9.1).

**Conclusions:**

We report a very low occurrence of community-acquired bacteraemia amongst COVID-19 patients in comparison to influenza patients. These results reinforce current clinical guidelines on antibiotic management in COVID-19, which only advise utilization of antibiotics when a bacterial co-infection is suspected.

## Introduction

In December 2019, the Wuhan Municipal Health Commission in China reported a few cases of pneumonia with an unknown aetiology. In these cases, the novel severe acute respiratory syndrome coronavirus 2 (SARS-CoV-2), was discovered [1, 2]. On February 27, 2020, the first case of coronavirus disease 2019 (COVID-19) was reported in the Netherlands [3]. After this first case, the SARS-CoV-2 virus spread rapidly in the south of the Netherlands. The southern region emerged as the Dutch epicentre of the first wave of COVID-19, putting a large strain on the microbiology laboratories in this area. Numerous analyses of SARS-CoV-2 tests, blood cultures and respiratory samples were performed. It was observed that during this period very few blood cultures were positive as a result of relevant bacteraemia, especially in comparison to blood cultures collected during influenza seasons. This raised questions about the occurrence of bacteraemia and bacterial co-infections in COVID-19 patients.

Bacterial co-infections are frequently identified in influenza infections and are an important cause of morbidity and mortality [4, 5]. It is estimated that 11–35% of all patients with an influenza infection acquire a bacterial co-infection [6]. The most frequently isolated pathogens are *Streptococcus pneumoniae*, *Staphylococcus aureus* and *Haemophilus influenzae* [7]. Although the use of empiric antibiotics is only recommended when a bacterial co-infection is suspected, it is difficult for physicians to differentiate between a viral infection and bacterial co-infection [8, 9]. This contributes to a widely recognized overuse of antibiotics in patients with viral infections [6, 10, 11]. For COVID-19, little is known about the occurrence of bacterial co-infections and the causative pathogens. One small observational study in the Netherlands showed an overall co-infection rate of 16% in COVID-19 patients [12]. Gaining more knowledge on this subject is important, particularly when deciding on the appropriate antibiotic regimen. During the first wave of the COVID-19 pandemic, many patients received empiric antibiotics prior to and during their admission to the hospital [13]. Numbers range from 31% in the Netherlands to 95% in Asia [3, 14]. Cephalosporins and broad-spectrum penicillins were most frequently prescribed in the Netherlands [13]. Unnecessary use of antibiotics has many disadvantages, especially in the view of a rising rate of antibiotic resistance [15].

Studying the occurrence, causative microorganisms and outcome of bacteraemia in COVID-19 patients in comparison to influenza A and B patients will increase the knowledge about bacterial co-infections in COVID-19 and possibly refine the current guidelines on antibiotic management.

## Methods

### Study design and population

We performed a retrospective cohort study of patients with an influenza A, influenza B or a COVID-19 diagnosis and analysed blood culture outcomes. Patients were enrolled from two different hospitals in the Netherlands, the Jeroen Bosch Hospital in ‘s-Hertogenbosch and Bernhoven Hospital in Uden. Patients were divided into three cohorts according to their infection status: (1) patients with an influenza A diagnosis in the influenza season 2015/2016 or 2016/2017, (2) patients with an influenza B diagnosis in influenza season 2017/2018 and (3) patients with a COVID-19 diagnosis between 28 February 2020 and 2 June 2020.

Patients were included when an influenza or a COVID-19 infection was confirmed by reverse transcription polymerase chain reaction (RT-PCR) on RNA from oropharyngeal swabs (in case of influenza) or oronasopharyngeal swabs (in case of COVID-19). Blood cultures were collected within a time interval of 48 h before and after the RT-PCR test.

Included into our analysis were patients attending the emergency department, patients diagnosed with influenza or COVID-19 at outpatients clinics who were subsequently hospitalized and patients who developed fever or flu-like symptoms during early hospitalization for other medical reasons. Patients who were discharged from the emergency department were analysed as well.

The following information was recorded and collected in an anonymous database: demographic data, viral diagnosis, blood culture results, names of isolated bacteria, likelihood of contamination, the hospital ward where the blood culture was collected, blood culture collection date and time and outcome of hospitalization expressed as 30-day all-cause mortality. Predominantly the data were extracted from the laboratory information system (MOLIS). Only the outcome of hospitalization was manually collected from the patient’s medical record. Bacteria were categorized as likely contaminants if they were affiliated to groups that represent commensal skin microbiota and were defined in the patient’s medical record as a contaminant by the Department of Medical Microbiology [16].

The percentage of positive blood cultures served as our primary outcome. Secondary outcomes were the percentage of relevant blood cultures, causative microorganisms and 30-day all-cause mortality.

This study was performed in line with the principles of the Declaration of Helsinki. The board of directors of both the Jeroen Bosch Hospital and Bernhoven Hospital approved this study. Additional ethical approval was granted by the Medical Ethical Review Committee Brabant (METC Brabant). Due to the collected data being held anonymously, informed consent was waived by the scientific review boards of both the Jeroen Bosch Hospital and Bernhoven Hospital for this study. Patients who had stated an objection to utilization of their medical data for scientific research in their electronic medical record were excluded from this study.

### Data analysis

Categorical variables are displayed as frequencies and percentages. Means and standard deviations were used to report continuous variables. The categorical variables were compared using the chi-square test or the Fisher’s exact test. The continuous variables were compared using a one-way analysis of variance (ANOVA). A *p* value < 0.05 was considered statistically significant. All analyses were performed with SPSS version 26.0 for Windows.

## Results

### Study characteristics

We examined 1635 patients who had blood cultures analysed and a diagnosis of either influenza A, influenza B or COVID-19. Some patients appeared multiple times in the list extracted from the laboratory information system due to numerous positive tests for one virus obtained during hospitalization. These patients were included only once, at the time of presentation to the hospital. Other patients were excluded due to blood cultures not collected within 48 h of viral testing. In the end, a total of 1324 patients were included. Seven patients were included twice due to being hospitalized with two different viral infections during various viral seasons. Therefore, the total number of viral episodes amounted to 1331. Among these, there were 325 influenza A infections, 328 influenza B infections and 678 COVID-19 infections. Blood cultures were collected at the emergency department in 88.2% of all viral episodes. There were no differences between the cohorts regarding median age. The frequency of males was higher in the influenza A cohort (58.2%) and in the COVID-19 cohort (65.1%). Detailed demographics are listed in Table 1.

**Table 1:** Demographics of patients with influenza A, influenza B or COVID-19

	Influenza A (***n*** = 325)	Influenza B (***n*** = 328)	COVID-19 (***n*** = 678)
Gender, n (%)			
Male	189 (58.2)	162 (49.4)	443 (65.1)
Age (years), median (interquartile range)	71 (59–81)	74 (64–83)	70 (58–78)
Age categorized (years), n (%)			
< 40	29 (9.0)	20 (6.1)	19 (2.8)
40–60	56 (17.2)	42 (12.8)	179 (26.3)
> 60	240 (73.8)	266 (81.1)	483 (70.9)
Presentation, n (%)			
Emergency Department	292 (89.8)	272 (82.9)	613 (90.4)
Outpatient clinic	1 (0.3)	1 (0.3)	4 (0.6)
Nursing ward	31 (9.5)	52 (15.9)	55 (8.1)
Intensive care	1 (0.3)	3 (0.9)	6 (0.9)

### Bacteraemia and clinical outcomes

There was no statistically significant difference (p = 0.47, Table 2) in the occurrence of blood culture positivity in influenza A patients (11.4, 95% CI 7.9–14.8), influenza B patients (10.4, 95% CI 7.1–13.7) and COVID-19 patients (9.0, 95% CI 6.8–11.1). After correcting for likely contaminants, the occurrence of relevant bacteraemia in COVID-19 patients declined to 1.0% (95% CI 0.3–1.8), which was statistically significantly lower (*p* = 0.04) than in influenza A patients (4.0, 95% CI 1.9–6.1) and influenza B patients (3.0, 95% CI 1.2–4.9). COVID-19 patients with relevant bacteraemia were significantly older (*p* = 0.03, Table 3) in comparison to patients without bacteraemia. No significant difference in gender was found in COVID-19 patients with relevant bacteraemia and patients without bacteraemia. The overall 30-day all-cause mortality rate was significantly higher (p = <.001, Table 4 in patients with COVID-19 (28.3, 95% CI 24.9–31.7) compared to patients with influenza A (7.1, 95% CI 4.3–9.9) and patients with influenza B (6.4, 95% CI 3.8–9.1). When examining the 30-day mortality in patients with positive blood cultures, a significantly higher mortality (*p* = 0.02, Table 4) was found in patients in the influenza A cohort (18.9, 95% CI 6.3–31.5) and patients in the COVID-19 cohort (26.2, 95% CI 15.2–37.3) compared to patients in the influenza B cohort (2.9, 95% CI − 2.7-8.4). There was no statistically significant difference in 30-day all-cause mortality between the different cohorts among patients with relevant bacteraemia.

**Table 2:** Proportion of positive blood cultures in patients with influenza A, influenza B or COVID-19

	Influenza A (***n*** = 325)		Influenza B (***n*** = 328)		COVID-19 (n = 678)		
	% (n)	95% CI	% (n)	95% CI	% (n)	95% CI	p value**
Positive blood cultures	11.4 (37)	7.9–14.8	10.4 (34)	7.1–13.7	9.0 (61)	6.8–11.1	0.47
Relevant positive blood cultures*	4.0 (13)	1.9–6.1	3.0 (10)	1.2–4.9	1.0 (7)	0.3–1.8	0.04

* Corrected for contamination

** Pearson’s chi-squared test

95% CI = 95% confidence interval

**Table 3:** Demographics of COVID-19 patients with relevant bacteraemia and patients without bacteraemia

	Relevant bacteraemic patients (***n*** = 7)	Non-bacteraemic patients (***n*** = 671)	p value
Gender, n (%)			
Male	4 (57.1)	438 (65.3)	0.72*
Age (years), median (interquartile range)	83 (70–85)	70 (58–78)	0.03**

* Pearson’s chi-squared test

** One-way ANOVA

**Table 4:** The 30-day all-cause mortality rate in patients with influenza A, influenza B or COVID-19 stratified by blood culture results

	Influenza A		Influenza B		COVID-19		
Mortality rate	% (n)	95% CI	% (n)	95% CI	% (n)	95% CI	p value
Overall	7.1 (23)	4.3–9.9	6.4 (21)	3.8–9.1	28.3 (192)	24.9–31.7	<.001**
With positive blood culture	18.9 (7)	6.3–31.5	2.9 (1)	-2.7-8.4	26.2 (16)	15.2–37.3	0.02**
With relevant positive blood culture*	30.8 (4)	5.7–55.9	10.0 (1)	−8.6-28.6	42.9 (3)	6.2–79.5	0.28***

* Corrected for contamination

** Pearson’s chi-squared test

*** Fisher’s exact test

95% CI = 95% confidence interval

### Causative microorganisms of bacteraemia

A total of 135 bacteria were isolated from 132 patients with positive blood cultures in the different cohorts. Of the 64 bacterial isolates in COVID-19 patients, 57 (89.1%) consisted of coagulase-negative staphylococci and other bacterial species that can be regarded as contaminants. This percentage of contamination was higher than in influenza A (64.9%) and influenza B patients (70.6%).

*Escherichia coli* and *S. pneumoniae* were the most common pathogens identified in patients with COVID-19, each accounting for 28.6% (*n* = 2) of bacteria causing a relevant bacteraemia. Other pathogens causing relevant bacteraemia in COVID-19 patients were: *S. aureus, Klebsiella pneumoniae* and *Pseudomonas aeruginosa* (all *n* = 1). *S. pneumoniae* was the most common cause of relevant bacteraemia among influenza A patients, causing 76.9% (*n* = 10) of all bloodstream infections in this cohort. The most frequently isolated bacteria in influenza B patients were *S. pneumoniae* and *S. aureus*, each accounting for 40.0% (*n* = 4) of all isolated bacteria. No bacteria seemed to be overrepresented in COVID-19 patients. The bacterial pathogens identified are further detailed in Tables 5 and 6.

**Table 5:** Bacteria isolated from positive blood cultures stratified by infection status

	Positive blood cultures		
Pathogen, n (%)	Influenza A (***n*** = 37)	Influenza B (***n*** = 34)	COVID-19 (***n*** = 61)
*Escherichia coli*	1 (2.7)	1 (2.9)	2 (3.1)
*Klebsiella pneumoniae*	1 (2.7)	–	1 (1.6)
*Pseudomonas aeruginosa*	–	–	1 (1.6)
*Micrococcus luteus*	1 (2.7)	–	–
*Streptococcus pneumoniae*	10 (27.0)	4 (11.8)	2 (3.1)
*Other Streptococcus* species*	1 (2.7)	1 (2.9)	1 (1.6)
*Staphylococcus aureus*	1 (2.7)	4 (11.8)	1 (1.6)
CNS	20 (54.1)	23 (67.7)	55 (85.8)
*Bacillus simplex*	1 (2.7)	–	–
*Brevibacterium casei*	–	1 (2.9)	–
*Corynebacterium* species	1 (2.7)	–	1 (1.6)
Total	37 (100)	34 (100)	64 (100)**

CNS = Coagulase-negative staphylococci

* *Streptococcus salivarius, Streptococcus pyogenes, Streptococcus vestibularis*

** Multiple bacteria were isolated from some of the blood cultures

**Table 6:** Bacteria isolated from positive blood cultures after correcting for contamination, stratified by infection status

	Positive blood cultures		
Pathogen, n (%)	Influenza A (***n*** = 13)	Influenza B (n = 10)	COVID-19 (***n*** = 7)
*Escherichia coli*	1 (7.7)	1 (10.0)	2 (28.6)
*Klebsiella pneumoniae*	1 (7.7)	–	1 (14.3)
*Pseudomonas aeruginosa*	–	–	1 (14.3)
*Streptococcus pneumoniae*	10 (76.9)	4 (40.0)	2 (28.6)
*Streptococcus pyogenes*	–	1 (10.0)	–
*Staphylococcus aureus*	1 (6.7)	4 (40.0)	1 (14.3)

## Discussion

Our study showed that only 1.0% of COVID-19 patients presenting at a general hospital experienced a clinically relevant bacteraemia, compared to 4.0% in influenza A patients and 3.0% in influenza B patients. *E. coli* and *S. pneumoniae* were most frequently isolated from positive blood cultures in COVID-19 patients. The same pathogens were also common in influenza A and B patients. We reported a 30-day all-cause mortality of 28.3% in COVID-19 patients, which was statistically significantly higher than the 30-day all-cause mortality of 7.1 and 6.4% found in respectively influenza A and B patients.

Our results are consistent with findings in a recent study of Sepulveda et al. [17]. They performed a large multicentre cohort analysis on patients with COVID-19 in New York. The authors reported a true bacteraemia rate of 1.6%. The most common causative microorganisms of bacteraemia in their study population were *E. coli*, *S. aureus*, *K. pneumoniae* and *Enterobacter cloacae* complex. A study of Hughes et al. also described similar findings [18]. The authors reported an occurrence of bacteraemia of 3.2%. This percentage consisted of both hospital- and community-acquired infections. We only reported on community-acquired infections, which may explain the difference. Their most frequently isolated community-acquired pathogens were *Enterobacterales, Streptococcus* spp., *S. aureus* and *Enterococcus* spp. A recent study of Goyal et al. noted a bacteraemia rate of 5.6% in COVID-19 patients [19]. This is significantly higher than the 1.0% that our study reported. Like Hughes et al., these authors examined both hospital-acquired and community-acquired infections, which may explain the higher rate of bacteraemia.

We reported a blood culture contamination rate of 89.1% amongst COVID-19 patients. This percentage is fairly consistent with other studies [17, 18]. This high percentage may be explained by the high workload and the recruitment of more inexperienced health care workers during the pandemic.

We found a 30-day all-cause mortality of 28.3% in our COVID-19 cohort. This number is relatively high compared to recent reports from Europe that describe 30-day all-cause mortality rates ranging from 19.7 to 21.5% [20, 21]. This disparity may be attributed to the higher risk of severe disease in our study population. During the first wave of COVID-19, family doctors in our region were asked to refer only severe cases to the overloaded hospitals. Furthermore, we only included patients with COVID-19 and analysed blood cultures. The required collection of blood cultures suggests these patients experienced severe febrile illness which indicates more severe disease.

Our findings on bacteraemia in influenza patients are consistent with prior studies [22, 23]. We reported a slightly lower rate of bacteraemia in influenza B patients in comparison to influenza A patients. This is in agreement with the assertion that influenza B often leads to milder disease and less co-infections when compared to influenza A [24, 25]. The 30-day all-cause mortality rate amongst influenza patients in our cohort was comparable to mortality rates mentioned in other literature [20, 26].

Our findings support current guidelines on antibiotic management that do not recommend the use of empiric antibiotics in patients with COVID-19 unless there is a clear suspicion of an accompanying bacterial infection [8]. However, differentiating between viral disease and a bacterial co-infection can be a challenge for physicians. Procalcitonin levels may be of assistance in determining the likelihood of bacterial co-infection and a potential adverse outcome [27]. When a bacterial co-infection is suspected and empiric antibiotics are administered, we advise to regularly collect blood cultures and respiratory samples. The antibiotic therapy may then be de-escalated or completely terminated based on the microbiological results. These recommendations will help in preventing overuse and the occurrence of potential side-effects from antibiotics.

A major strength of our study is the inclusion of over 1300 patients from two different hospitals in the Netherlands. This large sample size increases the accuracy and reliability of our results. In addition, we reported on community-acquired infections. Most studies on bacterial co-infections in COVID-19 failed to report on the setting of their study and possibly investigated hospital-acquired infections as well, which negatively affects the interpretability of their results [17, 28–30]. Finally, we did not experience any missing data, which minimizes the potential risk of information bias.

Some limitations should be mentioned. We did not collect data on prior antibiotic use, which may have led to an underestimation of the actual occurrence of bacteraemia in COVID-19 and influenza patients. Although the sample size of this study was of a reasonably large magnitude, the actual number of patients experiencing relevant bacteraemia was quite low, subgroup analyses of the 30-day all-cause mortality are therefore unreliable. In addition, we only reported on patients with bacteraemia and did not investigate other manifestations of bacterial co-infections. Thus, these results do not represent the entirety of bacterial co-infections in influenza and COVID-19 patients.

In conclusion, the proportion of clinically relevant community-acquired bacteraemia in COVID-19 patients was very low in comparison to influenza patients. These results justify the prudent use of empiric antibiotics in COVID-19 patients, when there is insufficient evidence of a bacterial co-infection.

## Data Availability

The datasets used and analysed during the current study are available from the corresponding author on reasonable request.
